# “Forever young at the table”: metabolic effects of eating speed in obesity

**DOI:** 10.1186/s12967-021-03199-1

**Published:** 2021-12-24

**Authors:** Luigi Barrea, Claudia Vetrani, Ludovica Verde, Bruno Napolitano, Silvia Savastano, Annamaria Colao, Giovanna Muscogiuri

**Affiliations:** 1 Department of Humanities, Pegaso Telematic University, 80143 Naples, Italy; 2 Italian Centre for the Care and Well-Being of Patients With Obesity (C.I.B.O), Department of Clinical Medicine and Surgery, Endocrinology Unit, Federico II University, Naples, Italy; 3 Department of Clinical Medicine and Surgery, Endocrinology Unit, Federico II University, Naples, Italy; 4 UNESCO Chair “Education for Health and Sustainable Development”, Federico II University, Naples, Italy

**Keywords:** Eating speed, Cardiometabolic diseases, Dyslipidaemia, Type 2 diabetes mellitus, Hypertension, Obesity

## Abstract

**Background:**

Cardiometabolic diseases (CMD) are recognized as the main causes of morbidity and mortality in developed countries. In recent years eating speed (ES) has been of particular interest since some studies have associated it with the development of obesity and CMD. However, the different impact of the ES at which main meals are eaten on the risk of developing these diseases has not yet been identified. Thus, we aimed to investigate the effect of ES at the main meals (breakfast, lunch, and dinner) on the risk of developing cardiometabolic diseases (type 2 diabetes mellitus, dyslipidaemia and hypertension) in middle-aged Caucasian subjects with obesity.

**Methods:**

For this purpose we carried out a cross-sectional, observational study. One hundred and eighty-seven middle-aged subjects aged 43.6 ± 16 years were enrolled of which anthropometric parameters and lifestyle habits were studied. A dietary interview was performed to collect information about meal duration and eating habits at the main meals. According to median value of meal duration, meals were classified in two groups: fast eating group (FEG) and slow eating group (SEG).

**Results:**

The prevalence of dyslipidaemia was more than twice in FEG compared to SEG at lunch and dinner. For all main meals, FEG had a significantly higher risk of dyslipidaemia than SEG (p < 0.05) in unadjusted model. However, when the model was adjusted for age, BMI, physical activity, smoking and alcohol use and medication, the result remained significant for lunch and dinner (p < 0.05).

**Conclusion:**

The results of our study suggest that fast eating increases at lunch and dinner increase the risk of developing dyslipidaemia in obesity.

## Introduction

Cardiometabolic diseases (CMD) are recognized as the main causes of morbidity and mortality in developed countries [1]. Lifestyle habits such as smoking, physical inactivity, and unhealthy dietary composition have been identified as modifiable risk factors for CMD [2–4]. Indeed, most of these unhealthy behaviours are associated with an increased oxidative stress that is known to have detrimental effects on cardiovascular system [5, 6]. However, in recent years also chrononutrition have shown to influence the risk of developing CMD [7]. In particular, it has been highlighted that the number and/or the time and/or the eating speed (ES) of meals might influence the risk of developing abdominal obesity and thus obesity related CMD [8–10]. Indeed, the results of a metanalysis carried out on 22 studies that experimentally manipulated ES highlighted that slower eating led to a significant reduction in food intake [10]. Conversely, fast eating was associated with an increased food intake [10]. Indeed, foods that can be consumed quickly and with distraction of attention from eating undermine the body’s capacity to regulate food intake at healthy levels [11]. Similarly, another metanalysis carried out on 23 studies highlighted the impact of fast eating on Body Mass Index (BMI) values [9]. In particular, the mean difference in BMI between individuals who ate quickly and those who ate slowly was 1.78 kg/m^2^ (95% confidence interval (CI), 1.53–2.04 kg/m^2^). The pooled odds ratio (OR) of obesity in fast eating individuals was 2.15 (95% CI 1.84–2.51) [9].

The physiopathological mechanisms behind this association may lie in the fact that the ES may influence several hormones involved in both the regulation of satiety and metabolism, i.e., insulin, glucagon-like peptide 1 (GLP-1), cholecystokinin, peptide YY (PYY) and pancreatic polypeptide [12–14]. In agreement with this hypothesis, a cross-sectional study carried out in 2704 males (mean age and BMI: 48.2 years and 23.3 kg/m^2^) and 761 females (46.3 years and 21.8 kg/m^2^) non-diabetic Japanese civil servants found that BMI correlated with ES in both sexes, and with daily energy intake in males [15]. In addition, in the very same study it has been reported a statistically significant parallel increase in insulin resistance (evaluated by HOMA-IR) with increases in ES in both genders, also when adjusting for age, energy intake and lifestyle factors. However, after adjusting for BMI, this positive relationship was confirmed only in males [15]. Fast eating has been also associated with significantly increased OR for high glucose and low HDL-cholesterol levels in males, even after adjusting for BMI [16]. Although the above reported studies highlighted the association of ES with obesity and CMD, they did not investigate if this association may vary according to the three different main meals (breakfast, lunch, and dinner).

Therefore, the purpose of this cross-sectional study was to investigate the effect of ES at the main meals (breakfast, lunch, and dinner) on the risk of developing CMD (T2DM, dyslipidaemia and hypertension) in middle-aged Caucasian subjects with obesity.

## Methods

### Design and setting

This cross-sectional, observational study was carried out in both patients attending the Endocrinology/Obesity Outpatient Clinic of Federico II University Hospital (Naples, Italy), students, non-medical employees, and participants in the Obesity, Programs of nutrition, Education, Research and Assessment of the best treatment (OPERA) project [17] from November 2020 to June 2021. The study was approved by the Ethical Committee of Federico II University (n.5/14) and conducted in accordance with the World Medical Association Code of Ethics (Declaration of Helsinki) for human experimentation. The aim of the study was clearly explained to all study participants and written informed consent was obtained. Recruitment consisted of an informational interview in which the details of the research were explained to the subjects, and they were encouraged to participate in the study.

### Participants

One hundred and eighty-seven middle-aged subjects aged 43.6 ± 16 years were enrolled in this cross-sectional study. Eligible participants for the study were adult subjects aged 18–75 years with normal liver, cardiopulmonary and kidney function as determined by interview. In addition, we excluded subjects taking medications for hepatic, renal and cardiopulmonary diseases. Trained nutritionists assessed anthropometric parameters, asked standard questions including demographic informations, personal medical history and lifestyle habits. Use of specific treatment for dyslipidaemia, T2DM and hypertension was also recorded.

### Anthropometric assessment

Subjects wore light clothing and no shoes while anthropometric parameters were measured, as previously reported [18, 19]. The formula for BMI was as follows: weight in kilograms divided by height in meters squared. A wall mounted stadiometer was used to determine height. A calibrated scale was used to determine body weight. Waist circumference (WC) was measured to the nearest 0.1 cm using a non-stretch tape measure at the natural indentation or at a midway level between lower edge of the rib cage and iliac crest if no natural indentation was visible, in according to the National Center for Health Statistics [20]. Grade I obesity was defined as a BMI between 30 and 34.9 kg/m^2^, grade II obesity as a BMI between 35 and 39.9 kg/m^2^, and grade III obesity as a BMI equal to or greater than 40.0 kg/m^2^ [21].

### Lifestyle habits

We defined individuals who smoked at least one cigarette per day as current smokers, former smokers were individuals who had quit smoking at least 1 year before the survey, and nonsmokers were the remaining participants. Participants who regularly exercised at least 30 min per day (YES/NO) were defined as physically active, as we have reported in detail in a previous study [22].

### Nutritional and eating speed assessment

As we have reported in detail in previous studies [23–26], dietary assessments were collected through a face-to-face interview with a qualified nutritionist. Since the ES could potentially have a different impact on metabolism throughout the day, the metabolic effect of ES will be assessed for each main meal (breakfast, lunch and dinner). A dietary interview was performed to collect information about meal duration (minutes) and eating habits (habitual consumed foods and beverages) at the main meals (breakfast, lunch, and dinner). According to median value of meal duration, meals were classified in two groups based on the following criteria: fast eating group (FEG) (breakfast < 10 min, lunch < 20 min, and dinner < 20 min) or slow eating group (SEG) [27] (breakfast ≥ 10 min, lunch ≥ 20 min, and dinner ≥ 20 min).

### Statistical analysis

Continuous variables with normal distribution were reported as mean ± SD while categorical variables were expressed as frequency or percentage. Difference between groups were tested by one-way ANOVA and by chi-square test for categorical variables. Logistic regression analyses were conducted to evaluate associations between ES and the presence of diabetes, dyslipidaemia and hypertension. Data were adjusted for age, BMI, gender, physical activity, smoking and alcohol use, and medication. Slow eating group was designated as the reference in all cases for ease of comparability. The p values were considered significant at p < 0.05 with 95% confidence interval. Statistical analysis was performed according to standard methods using the Statistical Package for Social Sciences software 26.0 (SPSS/PC; SPSS, Chicago, IL, USA).

### Sample size

In the absence of similar clinical studies available in the literature, the calculation of the sample size was performed a priori by considering the effect size 0.8 with type I error of 0.05 and a power of 90%. The number of subjects to be enrolled was found to be 34 per group. Since this sample not only met at least the necessary number of subjects, but also further supported the results, we decided to include all subjects in the statistical analysis. The calculation of the sample size was performed using G Power Software.

## Results

### Clinical characteristics of the study participants

The main clinical characteristics of the study population are reported in Table 1. One-hundred and eighty-seven participants (70 men and 117 women) were included in the analyses. They were aged 43.6 ± 16 years and presented a mean BMI 31.5 ± 7.5 kg/m^2^. Mean WC of enrolled subjects was 98.8 ± 21 cm while mean waist to hip ratio (WHR) was 0.91 ± 0.1. Most of the participants were sedentary (147, 78%), non-smokers (171, 91%) and no-alcohol consumers (170, 91%). The prevalence of CMD was as follows: 8 (4%) subjects with T2DM, 8 (4%) with hypertension and 17 (9%) with dyslipidaemia.

**Table 1:** Main characteristics of the study population

Parameters	Subjects (n = 187)
Gender (M/F)	70/117
Age (years)	43.6 ± 16
BMI (kg/m^2^)	31.5 ± 7.5
Waist circumference (cm)	98.8 ± 21
Hip circumference (cm)	108 ± 15
Waist/Hip ratio	0.91 ± 0.1
Physical activity	40 (22%)
Alcohol use	17 (9%)
Smoking	16 (9%)
Type 2 diabetes	8 (4%)
Hypertension	8 (4%)
Dyslipidaemia	17 (9%)

Data are expressed as mean ± SD or n (%)

### Clinical characteristics according to eating speed at meals

Tables 2, 3 and 4 show the main characteristics of the entire study population according to the ES at breakfast, lunch and dinner, respectively. No significant differences were observed in terms of gender proportion, age, anthropometric parameters and the prevalence of T2DM and hypertension between FEG and SEG at breakfast, lunch and dinner. Interestingly, the prevalence of dyslipidaemia was more than twice in FEG compared to SEG at dinner describing a p value (0.055) close to but not quite statistically significant as supporting a trend toward statistical significance. Furthermore, the qualitative analyses of foodstuffs consumed at the main meals did not highlight significant differences between the two groups (Table 5).

**Table 2:** Main characteristics of the whole study population according to eating speed at breakfast

Variables	Fast eating group (< 10 min) n = 83	Slow eating group (≥ 10 min) n = 93	p value*
Gender (M/F)	31/52	35/58	0.969
Age (years)	43.5 ± 16	40.7 ± 16	0.057
BMI (kg/m^2^)	32.0 ± 7	31.4 ± 8	0.612
Waist circumference (cm)	99.7 ± 18	99.2 ± 24	0.889
Hip circumference (cm)	109 ± 12	109 ± 14	0.723
Waist/Hip ratio	0.91 ± 0.1	0.90 ± 0.2	0.640
Type 2 diabetes	3 (4)	2 (28)	0.745
Dyslipidaemia	8 (10)	6 (6)	0.435
Hypertension	6 (7)	2 (28)	0.106

Data are expressed as mean ± SD or n (%). *p < 0.05 one-way ANOVA for continuous variables and χ^2^ test for categorical variables

**Table 3:** Main characteristics of the whole study population according to eating speed at lunch

Variables	Fast eating group (< 20 min) n = 124	Slow eating group (≥ 20 min) n = 63	p value*
Gender (M/F)	47/77	23/40	0.852
Age (years)	43.3 ± 16	43.8 ± 16	0.850
BMI (kg/m^2^)	31.7 ± 8	30.9 ± 7	0.532
Waist circumference (cm)	101 ± 22	97.6 ± 20	0.255
Hip circumference (cm)	109 ± 13	108 ± 13	0.803
Waist/Hip ratio	0.93 ± 0.1	0.89 ± 0.1	0.703
Type 2 diabetes	5 (4)	3 (5)	0.816
Dyslipidaemia	14 (12)	3 (5)	0.142
Hypertension	6 (5)	2 (3)	0.595

Data are expressed as mean ± SD or n (%). *p < 0.05 *vs* men, one-way ANOVA for continuous variables and χ^2^ test for categorical variables

**Table 4:** Main characteristics of the whole study population according to eating speed at dinner

Variables	Fast eating group (< 20 min) n = 108	Slow eating group (≥ 20 min) n = 79	p value*
Gender (M/F)	38/70	32/47	0.458
Age (years)	43.5 ± 17	43.4 ± 16	0.972
BMI (kg/m^2^)	32.0 ± 8	30.6 ± 7	0.194
Waist circumference (cm)	100 ± 22	98.1 ± 19	0.519
Hip circumference (cm)	109 ± 14	108 ± 12	0.504
Waist/Hip ratio	0.93 ± 0.1	0.89 ± 0.1	0.132
Type 2 Diabetes	5 (5)	3 (4)	0.781
Dyslipidaemia	12 (11)	5 (6)	0.055
Hypertension	6 (6)	2 (3)	0.261

Data are expressed as mean ± SD or n (%). *p < 0.05 one-way ANOVA for continuous variables and χ^2^ test for categorical variables

**Table 5:** Foodstuffs characterizing the main meals according to eating speed

	Fast eating group n (%)	Slow eating group n (%)
Breakfast		
Tea/herbal teas	9 (14)	3 (5)
Espresso coffee	29 (45)	1 (28)
Croissant	7 (11)	1 (28)
Semi-skimmed milk	7 (11)	11 (19)
Plant milk	–	2 (3)
Yogurt	2 (3)	–
Juice	2 (3)	4 (7)
Cookies	1 (28)	2 (3)
Protein pancakes	1 (28)	1 (28)
Fruit	1 (28)	1 (28)
Semi-skimmed milk and cookies	3 (5)	8 (14)
Semi-skimmed milk and breakfast cereals	1 (28)	10 (17)
Juice and cookies	–	5 (9)
The/tisane and cookies	–	8 (14)
Espresso coffee and cookies	1 (28)	1 (28)
Lunch		
Vegetable broth/consommé/stock	2 (28)	1 (28)
Cereal products	41 (48)	20 (49)
Cereal products and vegetables	10 (12)	7 (17)
Legumes	1 (1)	1 (28)
Vegetables	9 (10)	3 (7)
Bread and cured meat	8 (9)	4 (10)
Full meal^†^	10 (12)	3 (7)
Cereals and legumes	2 (28)	1 (28)
Pizza/potatoes	2 (28)	1 (28)
Animal protein-based dish	1 (1)	–
Dinner		
Animal protein-based dish	40 (50)	22 (48)
Animal protein-based dish and vegetables	10 (13)	11 (24)
Animal protein-based dish and cereal products	4 (5)	–
Ultra-processed foods^††^	3 (4)	3 (7)
Bread and cured meat	2 (3)	1 (28)
Cereal products and vegetables	8 (10)	–
Pizza/potatoes	9 (11)	3 (7)
Vegetables	5 (6)	6 (13)

^†^Full meal consists in cereal-based mean course, animal protein-based dish, vegetables, and fruit

^††^Cordon bleu, fish sticks, frozen salty soft dough with ham and cheese

### Association of eating speed with the risk of T2DM, dyslipidaemia, and hypertension

**Fig. 1:**
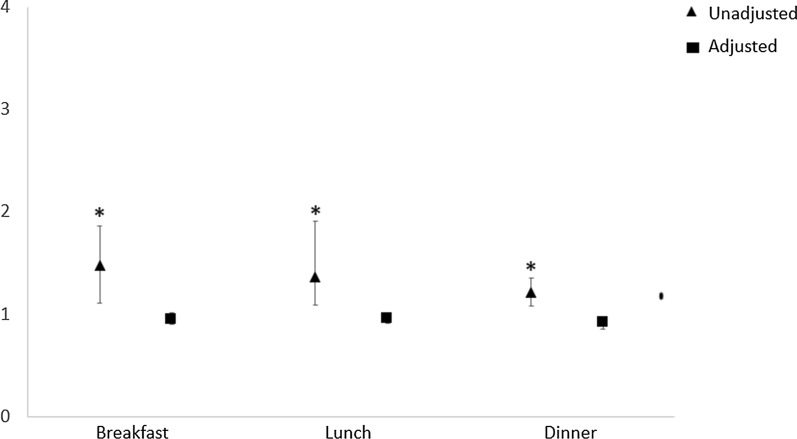
Logistic regression analyses for type 2 diabetes risk by eating speed at the main meals. Data were adjusted for age, BMI, gender, physical activity, smoking and alcohol use, and medication (except for antidiabetic drugs). *p < 0.05

**Fig. 2:**
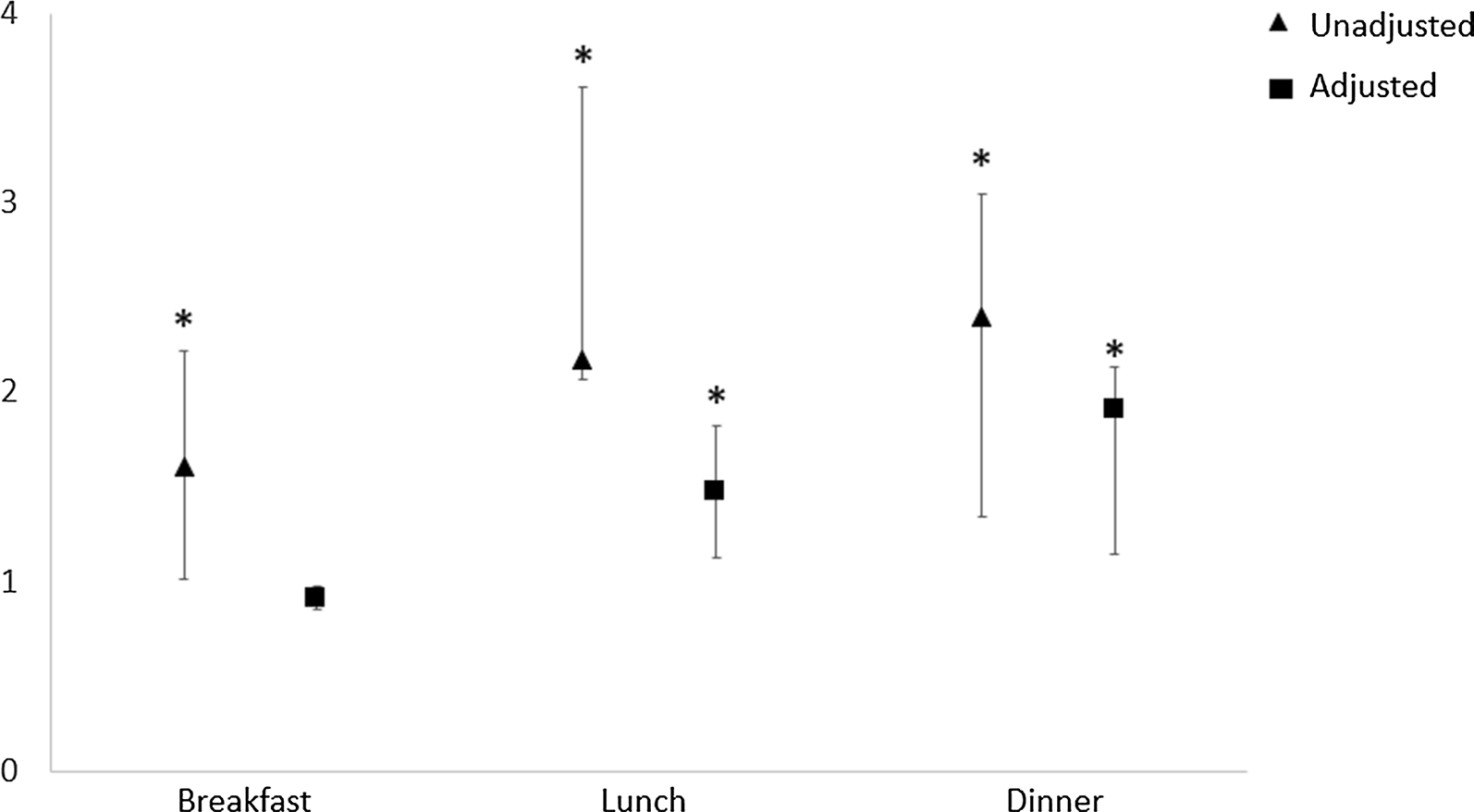
Logistic regression analyses for dyslipidaemia risk by eating speed at the main meals. Data were adjusted for age, BMI, gender, physical activity, smoking and alcohol use, and medication (except for antilipemic drugs). *p < 0.05

**Fig. 3:**
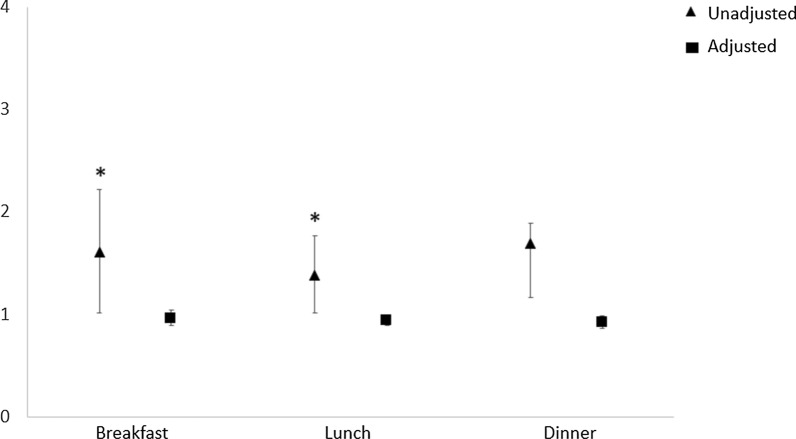
Logistic regression analyses for hypertension risk by eating speed at the main meals. Data were adjusted for age, BMI, gender, physical activity, smoking and alcohol use, and medication (except for antihypertensive drugs). *p < 0.05

Logistic regression analysis showed that, for all main meals (breakfast, lunch and dinner), FEG showed a significantly higher risk of T2DM than SEG in the unadjusted model (p < 0.05), but this association was no longer significant in the adjusted model for age, BMI, physical activity, smoking and alcohol use, and medications (Fig. 1). About the risk of dyslipidaemia, FEG had a significantly higher risk of dyslipidaemia than SEG (p < 0.05) in unadjusted models for all the main meals. When the model was adjusted for all the confounding factors the result remained significant only for lunch (p < 0.05) and dinner (p < 0.05) (Fig. 2). Finally, FEG showed a significantly higher risk of hypertension than SEG at breakfast and lunch in the unadjusted model (p < 0.05), that was lost in the adjusted model (Fig. 3).

## Discussion

The main purpose of this study was to investigate the effect of ES at the main meals (breakfast, lunch and dinner) on the risk of developing CMD in middle-aged Caucasian subjects with obesity. Although fast eating at breakfast, lunch and dinner was significantly associated to the risk of developing T2DM, hypertension and dyslipidaemia, these associations were lost in a model adjusted for all confounding factors (age, BMI, gender, physical activity, smoking and alcohol use, and medication) except for the risk of developing dyslipidaemia that was significantly associated to fast eating at lunch and dinner.

In agreement with our findings, the relationship between fast eating and lipid abnormalities, such as increased plasma triglycerides and decreased plasma HDL concentration, has also been highlighted previously. For instance, Paz-Graniel et al. carried out a study (N = 792), where the ES was assessed trough a non-validated ad-hoc eating behavior questionnaire that included information about how they perceived their ES in the main meals classified in 5 categories (very slow, relatively slow, medium, relatively fast, very fast), reporting a 59% higher risk of hypertriglyceridemia in the very fast eating group compared with the slow (both very and relatively slow) eating group [28]. In accordance with this findings, Tao L. et al. (N = 7972) report that increasing ES (assessed by asking “How fast is your speed of eating?” and with three self-reported response options, slow, normal and fast) was associated with a high risk for elevated triglycerides and with a reduction in HDL-cholesterol [29]. Likewise, Nagahama et al. (N = 56,865) found out that fast eating (assessed by asking “How fast is your speed of eating?” and with three self-reported response options, slow, normal and fast) was associated with increased odds of abnormal lipid profiles in men [30]. In a study conducted in 30 non-alcoholic, non-diabetic, overweight women, ES (evaluated by an eating monitor tool) positively correlated with waist-hip circumference values and triglyceride levels [31] whereas in a cross-sectional analysis carried out in Korean men, an inverse relationship was found between ES (classified as follow: < 5 min per meal, between 5 and 10 min per meal, between 10 and 15 min per meal and > 15 min per meal) and HDL-cholesterol levels [16]. Finally, in a multiethnic Asian population in which ES was assessed as self-reporting, faster eaters had significantly higher blood pressure, circulating triglycerides, and total to HDL-cholesterol ratios than slower eaters [32]. As far as hypertriglyceridemia is concerned, some authors suggested that the intake of a high amount of calories over a short frametime induces more sustained peaks in plasma glucose and insulin [15, 33], which could favor an anabolic state that stimulates liver lipogenesis and therefore increases plasma triglyceride levels [31, 34, 35].

The potential mechanism by which ES has an impact on the risk of developing CMD is still unclear. Eating is a complex physiological act influenced by numerous endogenous and exogenous factors [36]. In recent years, knowledge of the mechanisms involved in appetite control has increased, and the immediate postprandial state is now thought to be characterized by hormonal changes that include a decrease in concentrations of the intestinal orexigenic peptide ghrelin and concomitant increases in anorexigenic peptides such as PYY and GLP-1 [37]. Ghrelin, PYY, and GLP-1 act on the hypothalamus and play an important regulatory role in the mediation of hunger, satiety, and energy intake [37] but also in the regulation of body weight and lipid metabolism [38, 39]. In this respect, the ES also seems to play a part in the modulation of these physiological mechanisms, and very recent data from the literature support an association between ES and levels of PYY and GLP-1, two important players in this context. For instance, in a study carried out in young males (lean subjects aged 20.8 ± 0.8 years; subjects with obesity aged 20.4 ± 0.7 years), subjects with obesity (N = 14), had a fast ES rate and a lower number of chews per 1 gr of food compared with lean ones (N = 16), [40]. In addition, the authors investigated the effect of fast (15 chews) and slow ES (40 chews) on gastrointestinal hormones in all cohort. They found that slow ES resulted in lower energy intake and postprandial ghrelin concentration along with higher postprandial GLP-1 and cholecystokinin concentrations in both lean and subjects with obesity compared to fast ES [40]. In another study with a cross-over design (N = 17), a higher postprandial PYY and GLP-1 levels were found after slow ES (30 min) compared to fast ES (5-min meal), but no effect on ghrelin suppression [13]. In agreement with these data a study carried out in overweight adolescents (N = 27) who used a mandometer (a device that provides real-time feedback on meal consumption by plate weight) to slow ES compared with the control group found that slowing ES resulted in an increased PYY response and greater ghrelin suppression postprandially [41]. Thus, it is therefore possible to speculate that fast ES elicits a weaker anorexigenic gut hormone response despite there is currently no consensus on the effect of ES on the satiety hormone response and further research is needed.

In addition, interesting data are arising on the modulation of lipid metabolism by GI hormones. Evidence in beginning to accumulate that GLP-1 have direct effect on lipid metabolism. GLP-1 mediates its effects by binding to its receptor, the GLP-1 receptor (GLP-1R), abundantly present in the pancreatic beta cells, gut, and the central nervous system and moderately in the lung, heart, kidney, blood vessels, pancreatic alpha cells, and peripheral nervous system [42]. GLP-1R also have been found in adipose tissue [43]. In human adipocytes, GLP-1 exerts a dual action, depending upon the dose, on lipid metabolism, being lipogenic at low concentrations of the peptide, and lipolytic on at doses 10–100 times higher; both effects are time and GLP-1 concentrations dependent [39]. Moreover GLP-1 would appear to increase the elimination of fatty acid deposits in hepatocytes through increased FFA flux, increased oxidation and autophagy [44]. Similarly, PYY also appears to play a part in the regulation of lipid metabolism. Indeed, PYY was able to regulate specific transcription factors (specifically, RXRa e PPAR, implicated in the production of chylomicrons as well as the biogenesis of apos B-48 and B-100) impacting in the regulation of lipid and cholesterol metabolism [45]. Recent studies have reported an inverse correlation between fasting PYY and total cholesterol [46], as well as low- and high-density lipoprotein cholesterol levels [47], which indicates that the PYY gut hormone may be involved in the modulation of cholesterol metabolism. In post-prandial state PYY decrease cholesterol absorption by reducing the protein mass of NPC1L1, a protein essential for the absorption of dietary cholesterol [45]. Thus, if it has been reported that slow ES increase post-prandial PYY, thus resulting in a better lipid metabolism.

In addition, as previously reported circadian variations in GLP-1 secretion have been described [48–51] with increases in plasma concentrations after the mean 3 meals, consistently high throughout the day, falling to fasting levels only at night [52]. The secretory rhythm of PYY is also influenced by meals, with high postprandial concentrations and a peak after lunch [53]. Thus, we observed that the risk of developing dyslipidaemia was more associated to lunch and dinner instead of breakfast because at these times of the days (lunch and dinner) it is expected that GLP-1 and PYY would be at lower levels than in the morning due to their circadian rhythm. Thus, fast ES at lunch and dinner could set PYY and GLP-1 at levels that would be lower than physiologically occurs.

Our study has several strengths. Firstly, this is the first study that investigated the effect of ES of the three main meals on the prevalence of CMD. Indeed, there are already evidence in the literature that investigating metabolic effect of eating speed of a meal but we decided to consider the difference of metabolic effects of three main meals of the day because of circadian variation in the secretion of gastrointestinal hormones. Thus, each meal of the day “meets” a different gastrointestinal hormonal “*picture*” and this could result in different metabolic effects. Second, the population was very homogeneous and therefore, the present results may be highly generalizable to clinical settings. Third, we adjusted the data for several confounding factors, including clinical characteristics such as age, gender and BMI and other lifestyle factors. The limitations of our study should also be noted. Our results are from a cross-sectional analysis and a causal relationship between ES and CMD can not be demonstrated. In addition, we had no data on meal sizes, so fast eaters could be so because they ate smaller meals than slower eaters. However, we did not observe any differences in BMI between the two groups, so it is likely that the faster eaters were eating meals of poorer quality and with a higher energy density, factors that could result in the worse metabolic profile observed. In fact, highly processed solid foods, semi-solid desserts and high-calorie beverages are foods often associated with a large bite or sip and inadequate oral processing due to reduced chewing of each bite [54]. This can result in a consequent increase in ES. In addition, ultra-processed foods also have a high energy density due to their sugar and fat content and this may also lead to an increase in calorie intake [54]. Finally, another limitation of the study is that it did not include a "chew count" for each mouthful of food.

In conclusion, our study highlights the importance of ES in the risk of developing dyslipidaemia in obesity thus pointing out the importance of assessing ES in obesity outpatient clinic. Dietary education on the benefit of slow eating could be an easy way to potentiate the beneficial effects of antiobesity treatment on cardiometabolic risk.

## Data Availability

Not applicable.

## Abbreviations

CMD: Cardiometabolic diseases
ES: Eating speed
BMI: Body Mass Index
T2DM: Type 2 diabetes mellitus
OR: Odds ratio
CI: Confidence interval
GLP-1: Glucagon-like peptide 1
GLP-1R: GLP-1 receptor
PYY: Peptide YY
WHR: Waist to hip ratio
